# Editorial: The application of immune checkpoint inhibitors combined with chemotherapy in tumor immunotherapy

**DOI:** 10.3389/fimmu.2025.1736267

**Published:** 2025-11-18

**Authors:** Yufei Wang, Chao Wang, Yueying Li, Hongfei Jiang

**Affiliations:** 1 Guangxi Key Laboratory of Special Biomedicine, School of Medicine, Guangxi University, Nanning, China; 2 The Affiliated Hospital of Qingdao University and Qingdao Cancer Institute, Qingdao, China; 3 Department of Medicinal Chemistry, College of Pharmacy, University of Michigan, Ann Arbor, MI, United States

**Keywords:** immune checkpoint inhibitors (ICIs), chemotherapy, tumor immunotherapy, combination therapy, biomarkers

## 1 Background and aims

**Figure 1:**
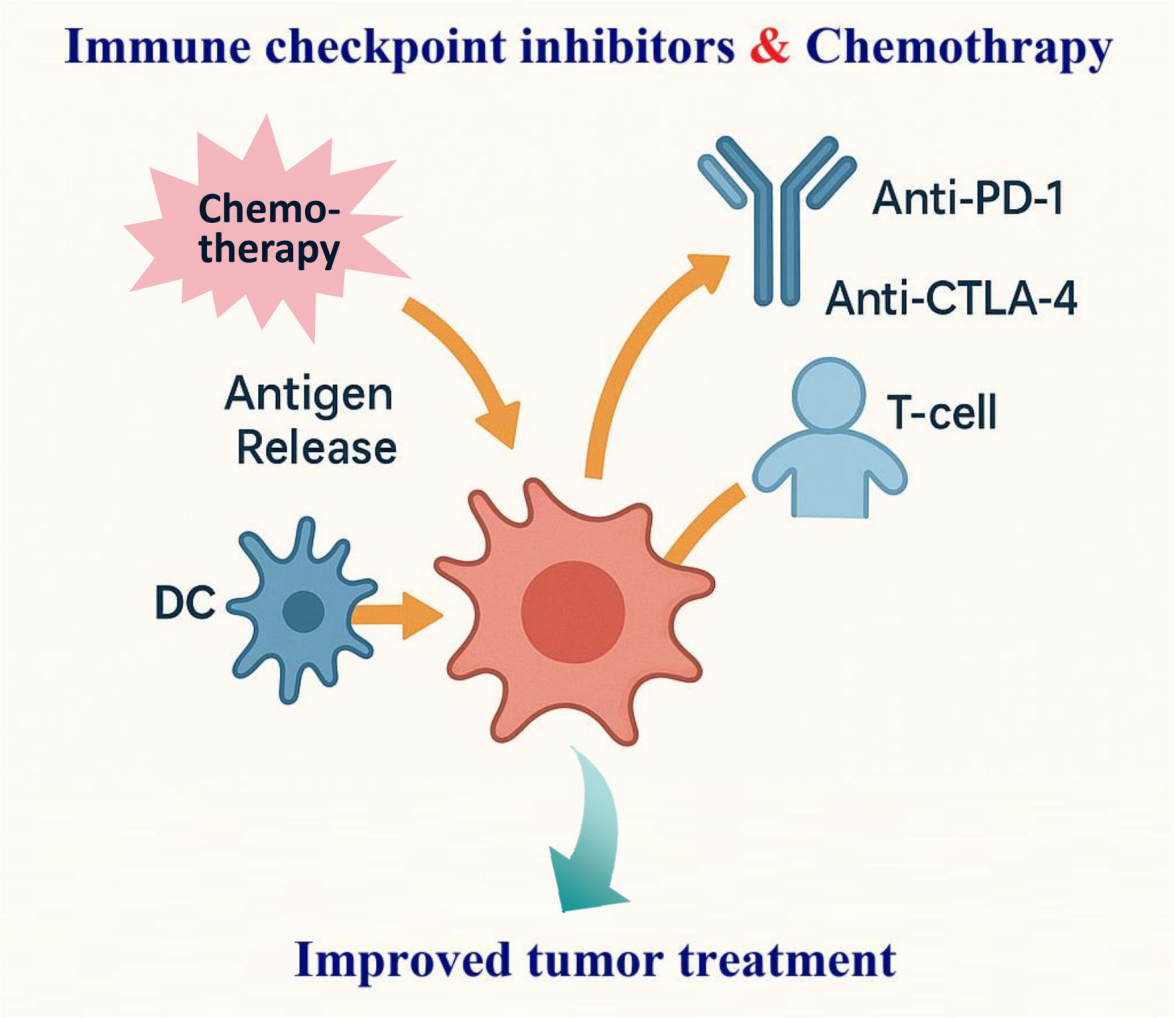
Schematic of synergistic mechanisms of immune checkpoint inhibitors (ICIs) combined with chemotherapy in tumor immunotherapy. Chemotherapy promotes antigen release, exposes DAMPs, primes dendritic cells (DCs), and suppresses immunosuppressive populations. ICIs (anti-PD-1/anti-CTLA-4) then better reinvigorate T cells enhancing.

Combining immune checkpoint inhibitors (ICIs) with chemotherapy has reshaped cancer care by pairing immune reinvigoration with tumour debulking and microenvironmental reprogramming. Cytotoxics can heighten antigen release, expose damage-associated molecular patterns, prime dendritic cells (DCs), and transiently curb immunosuppressive populations—creating a therapeutic window in which PD-(L)1 or CTLA-4 blockade is more effective (1, 2). Clinically, this biology has translated into higher response rates and survival gains across several solid tumours, exemplified in metastatic non-small-cell lung cancer where pembrolizumab plus platinum/pemetrexed became a first-line standard (3). Evidence also supports triplet strategies that add anti-angiogenic therapy to ICI–chemotherapy, improving progression-free and overall survival in nonsquamous disease (4). In curative-intent pathways, neoadjuvant ICI–chemotherapy deepens pathological regression and prolongs event-free survival in resectable NSCLC, anchoring a perioperative paradigm (**Figure 1**) (5). Beyond lung cancer, early triple-negative breast cancer demonstrates durable event-free survival benefits when pembrolizumab is integrated with neoadjuvant chemotherapy and continued post-operatively (6).

Amid these advances, key questions remain around deployment, selection, and safety: how to sequence therapy (induction vs concurrent), how many cycles are sufficient in the neoadjuvant setting, which composite or on-treatment biomarkers best guide patient selection, and how to preserve dose intensity while managing immune-related and cytotoxic adverse events. This Editorial distils the mechanistic rationale and current practice patterns of ICI–chemotherapy combinations and outlines a forward-looking agenda—standardized reporting of timing and dose intensity, integration of adaptive biomarkers for real-time triage, and rational escalation to triplets when biology supports it—to help clinicians design and deliver chemo–immunotherapy safely and effectively across tumour types.

## 2 Thematic landscape and key findings

The systematic integration of chemo–immunotherapy evidence across tumour types converges on three axes—sequencing, patient selection, and safety/feasibility—offering a practical scaffold for clinical use.

### 2.1 Lung cancer: from perioperative pathways to metastatic disease

Liu et al. synthesise neoadjuvant NSCLC trials in a Bayesian meta-analysis, showing that immuno-chemotherapy plus anti-angiogenic therapy ranks highest for pathological response, that ≤3 cycles likely capture most benefit, and that MPR outperforms pCR as a surrogate for event-based outcomes—directly informing perioperative design and endpoints.

Zhang B. et al. examine the administration sequence of ICIs and chemotherapy in advanced NSCLC and find discrete efficacy differences between sequences, arguing for sequence-aware prospective trials that align pharmacodynamic windows with immune priming.

Zhang et al. extend the mechanistic base: dual blockade of BTLA and PD-1 reverses CD8^+^ T-cell exhaustion in NSCLC, suggesting a rational platform for triplets layered on chemo-ICI in settings of adaptive resistance.

Together, these studies delineate a perioperative-to-metastatic evidence chain: optimise how we combine (cycle number and sequence), refine what we measure (MPR as a pragmatic surrogate), and expand with whom we intensify (mechanistically selected triplets).

### 2.2 Gastrointestinal tumours: from LARC to gastric/GEJ strategy refinement

Li et al. implement total neoadjuvant immuno-chemotherapy (iTNT) for pMMR/MSS LARC, across short-course radiotherapy pathways with different ordering, reporting encouraging clinical and pathological remissions with manageable toxicity—supporting watch-and-wait discussions under MDT oversight.

Zhao et al. provide a multi-institutional comparison of immunotherapy-based TNT versus standard chemoradiation in LARC, underscoring the feasibility and potential advantages of integrating immunotherapy while defining signals to test in randomized trials.

Cui et al. develop and externally validate a nomogram that predicts pCR after neoadjuvant immuno-chemotherapy in locally-advanced gastric cancer, enabling pre-operative risk-adapted decisions and pathway management.

Zhang et al. synthesise first-line PD-1-based chemo-combinations for advanced gastric/GEJ adenocarcinoma in a network meta-analysis, clarifying efficacy–toxicity trade-offs to guide regimen selection beyond single trials.

Collectively, these articles move GI practice from “whether to add ICI” to “how to personalise ICI-chemotherapy”—via risk tools (nomograms), pathway-specific TNT designs, and comparative syntheses that balance benefit with toxicity.

### 2.3 Real-world and case-based signals: extending to edge populations and complex scenarios

Jia et al. deliver a propensity-score–matched real-world analysis in ES-SCLC, confirming first-line chemo-ICI efficacy and safety relative to chemotherapy alone and helping bridge the gap between trials and routine practice.

Chen et al. report HER2-positive gastric cancer with bone-marrow metastasis and DIC achieving long-term survival on cadonilimab + trastuzumab + chemotherapy, with local radiotherapy and maintenance—illustrating how bispecific ICI plus HER2 targeting can rescue extremely high-risk disease.

Wang et al. describe advanced pulmonary large-cell neuroendocrine carcinoma managed with multi-line immuno-chemotherapy plus anti-angiogenic therapy, achieving nearly four-year OS and supporting combination strategies in this aggressive histology.

Zhu et al. present pulmonary adenosquamous carcinoma harbouring co-occurring drivers with a sustained complete response to ICI within a chemo-immuno framework, highlighting that molecular complexity need not preclude durable immune benefit.

Lang et al. show complete remission in high-risk locally-advanced cervical cancer treated with first-line tislelizumab + bevacizumab + chemotherapy, followed by radiotherapy and maintenance—an instructive multimodal sequence for difficult pelvic disease.

These real-world and case-based contributions extend the reach of chemo-immunotherapy to rare histologies, high-risk presentations, and multimodal pathways, while underscoring the importance of toxicity governance and sequencing in practice.

## 3 Conclusion and outlook

Taken together, the contributions in this Topic argue that chemo–immunotherapy is most effective when it is deliberately timed, sequence-aware, and risk-adapted rather than used reflexively. Across lung and gastrointestinal cancers—and even in edge populations—the evidence supports three pragmatic shifts: (i) optimise how we combine (limited neoadjuvant cycles, explicit induction vs. concurrent choices, and biologically justified maintenance); (ii) upgrade what we measure (adopt MPR as a practical perioperative surrogate, paired with pre-operative risk tools); and (iii) strengthen guardrails (plan for overlapping toxicities and report sequence, cycle number, dose intensity/density uniformly). This synthesis provides a transferable scaffold for trial design and day-to-day decision-making across tumour types.

Looking ahead, four priorities can make chemo–immunotherapy consistently durable and mechanism-anchored. First, build sequence-aware perioperative regimens that align chemotherapy-driven antigen release with checkpoint reinvigoration and predefine the role/duration of adjuvant therapy (informed by KEYNOTE-671, AEGEAN, CheckMate 77T) (7–9). Second, upgrade endpoints by embedding major pathological response within hierarchical/composite frameworks and quantifying its bridge to EFS/OS, so early signals translate into patient-centred benefit (7, 8). Third, steer escalation or de-escalation with multimodal biomarkers—functional T-cell states, histopathology, ctDNA kinetics, and imaging–pathology AI—to enable organ preservation where appropriate. Fourth, evaluate rational triplets judiciously when resistance biology is explicit, and anchor maintenance choices to adjuvant benchmarks (e.g., IMpower010) with uniform reporting of sequence, cycle number, dose intensity, and dose density for reproducibility (10).
